# Accuracy of iView and PIPSpro registration software

**DOI:** 10.1120/jacmp.v11i4.3180

**Published:** 2010-08-25

**Authors:** Stefania Pallotta, Marco Ceroti, Marta Bucciolini

**Affiliations:** ^1^ Dipartimento di Fisiopatologia Clinica Universita' di Firenze 50134 Firenze Italy; ^2^ U.O. Epidemiologia Molecolare e Nutrizionale ISPO 50134 Firenze Italy

**Keywords:** image registration, portal imaging, DRR, accuracy

## Abstract

In radiotherapy, treatment portal images and digitally reconstructed radiographs (DRRs) are used to monitor patient setup during clinical routine. The output of the registration between the portal image and the reference DRR indicates the patient displacement. If the registration is not reliable, the patient positioning will not be accurate. The aim of this work is to assess the intrinsic and the global accuracy of iView and PIPSpro, two widely used registration software programs that implement a manual and a semiautomatic approach, respectively. The intrinsic accuracy was tested using a computer generated phantom, while the overall accuracy was evaluated registering the portal images and the DRRs of an Alderson RANDO phantom. For DRRs, four treatment planning systems (TPS) and three CT studies with different slice thicknesses were considered.

This study demonstrates that the intrinsic accuracy of iView and PIPSpro were within 1 pixel and 1°. Using a DRR extracted from a 2 mm CT study, the overall accuracy of both methods was about 2 mm and 1°. When thicker CT slices are considered, the global accuracy of both methods worsens, and differences larger than 1.5° between the rotation parameters estimated with iView and PIPSpro are evident. The results obtained with iView and PIPSpro were nearly equivalent.

PACS numbers: 87.57.nj, 87.57.cp

## I. INTRODUCTION

A growing demand for accuracy in radiotherapy treatment makes imaging tools the key component in the entire procedure, from planning to treatment delivery. In particular, image‐guidance systems have been developed and employed such as: electronic portal imaging devices (EPIDs), surface scanners, ultrasound^(^ ^1^ ^–^ ^3^ ^)^ and cone beam CT.^(^ ^4^ ^,^ ^5^ ^)^

To monitor and correct patient setup, several kinds of registration techniques are used. 3D and 2D‐3D registration methods^(^ ^6^ ^)^ are used but have not yet been exploited as much as the 2D techniques. In fact, even though an accurate treatment is necessary for all patients, 3D monitoring and correction strategies are not always possible or appropriate in all situations.

For these reasons EPIDs are still the most frequently used image‐guidance systems to check patient setup during clinical routine. As a reference, both simulator images and digitally reconstructed radiographs (DRRs) can be used, even though DRR is widely recognized currently as the reference image.^(^ ^7^ ^)^

In this context, the accuracy of patient setup depends on the accuracy of the registration software^(^ ^8^ ^)^. The aim of the present study is to evaluate and compare the intrinsic and the overall accuracy of two widely used registration software programs, iView and PIPSpro. The intrinsic accuracy of the registration software – that is, the program's ability to match the actual registration parameter when these are a priori known – is investigated using a computer‐generated phantom. On the other hand, the overall accuracy that takes into account the imaging contribution to the registration procedure was studied using real phantom data.

CT studies with various slice thicknesses and four treatment planning systems (TPS) were considered for DRR generation.

## II. MATERIALS AND METHODS

### A. Registration software

iView (Elekta, Crawley, UK) is an image processing software developed to display and acquire images using iView and iViewGT portal imaging devices. It enables a manual registration between two images using an interactive template matching strategy based on a bi‐dimensional global rigid transformation (iViewGT User's Manual, Elekta, Crawley, UK). Templates representing treatment field contours and bone structures of the reference image are prepared by the user, overlapped on corresponding portal images and manually fitted until the match is considered satisfactory. Two translations and an in‐plane rotation, representing patient displacement with respect to the treatment field, are reported.

PIPSpro (Standard Imaging Inc., Middleton WI) is an image processing system developed specifically for quality control of EPIDs and for the automatic analysis of digital images and films. Besides standard routines for enhancement, display and analysis of digital images, three registration tools are provided. The algorithms, based on a bi‐dimensional global rigid transformation – template registration, chamfer matching^(^ ^9^ ^)^ and fiducial points (PIPSpro User's Guide, Masthead Imaging Corporation, Nanaimo, BC, Canada) – are also provided. In all cases, the registration parameters (two translations and an in‐plane rotation) represent patient displacement with respect to the treatment field. Before using any of the three methods, a set of corresponding fiducial points and/or contour templates must be defined in both images. While the template registration is a manual procedure similar to that described for iView, fiducial and chamfer options are semiautomatic methods. Only the fiducial points and chamfer matching method performances were evaluated in this study.

### B. Portal imaging and DRR

All portal images were acquired with a standard amorphous silicon EPID (iViewGT, Elekta, Crawley, UK) using a 6 MV photon beam from an Elekta Precise Treatment System. Portal images were acquired with the same exposure technique used for patient position verification.

DRR images were generated using four TPSs: XiO (CMS, St. Louis, MS), Pinnacle (Philips Radiation Oncology Systems, Mountain View, CA), Odyssey 3.6 (PerMedics Inc, San Bernardino, CA), Ocentra MasterPlan 1.4 (Nucletron, Veenendaal, The Netherlands), and three CT studies with different slice thicknesses.

### C. Tests

#### C.1 Intrinsic accuracy of the registration software

**Figure 1:**
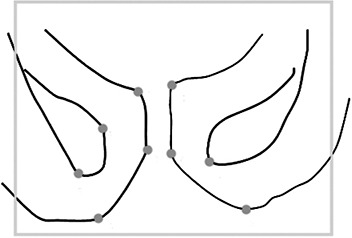
Synthetic phantom: the outline of the pelvis bone structures together with a rectangular treatment field edge (in grey) are used as reference image. Grey dots represent the fiducial points.

A synthetic phantom (700×700 pixels) representing a realistic template of pelvis bone structures (Fig. 1) was created to assess the intrinsic accuracy of the registration software. The bone structure outlines were rotated (range 0°–9°) and translated along both axes (range 1–15 pixels) thus resulting in nine different images (positions in Table 1). Each image was then overlapped on the field edge of the reference image, thus simulating the different patient displacements with respect to the treatment fields. The resulting images were registered on the reference image and the registration parameters were compared with the known displacement values. This procedure was repeated three times for each displacement. In order to test the different software performances, the mean value and the standard deviation of the differences between the known displacement values and the registrations' results were evaluated.

**Table 1:** Intrinsic accuracy of the registration software x, y and α are the a priori known values.$\bar{\Delta }x,\bar{\Delta }\pm$ and $\bar{\Delta }y$ are the mean differences between registration parameters and a priori known values.

			*iView*			*PIPS Chamfer*			*PIPS Fiducial*		
x *[pixel]*	y *[pixel]*	α *[°]*	**Δ*x*** *[pixel]*	**Δ*y*** *[pixel]*	**Δα** *[°]*	**Δ*x*** *[pixel]*	**Δ*y*** *[pixel]*	**Δα** *[°]*	**Δ*x*** *[pixel]*	**Δ*y*** *[pixel]*	**Δα** *[°]*
0	0	0	0	0	0	0.5	0.5	0	0.5	‐0.5	0
5	0	0	0	‐0.7	0	1.0	1.0	0	‐0.5	1.0	0
10	0	0	‐0.7	‐0.3	0	1.0	0.5	0	‐0.5	1.0	0
15	0	0	‐0.3	0	0	1.0	1.0	0	‐0.5	‐0.5	0
0	5	0	0.7	0.3	0	0.0	0.5	0	0.5	0.5	0
0	10	0	0.7	‐0.3	0	1.0	0.0	0	‐0.5	1.0	0
0	15	0	0	0.3	0	‐1.0	1.0	0	‐0.5	0.5	0
0	0	3	0	0	‐0.1	0	0	0	0	0	0
0	0	6	0	0	‐0.1	0	0	1	0	0	0
0	0	9	0	0	0.1	0	0	0	0	0	0

The bone structure outlines were used as template contour in both iView and chamfer matching options of PIPSpro. When the fiducial points option of PIPSpro was tested, nine reference points, represented by grey dots in Fig. 1, were used.

#### C.2 Overall accuracy

**Figure 2:**
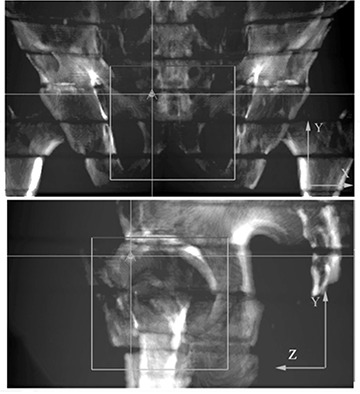
AP and LR DRR of the Alderson RANDO phantom with overlapped treatment fields obtained with 2 mm CT slice thickness and Odyssey TPS.

Three CT studies of the Alderson RANDO phantom (2 mm, 3 mm and 5 mm slice thickness; 0.78 mm pixel size) were acquired using a Somatom Plus 4 (Siemens AG, Munich, Germany) scanner. For each TPS and for each CT study a conventional four‐field box, with the same isocenter was applied, thus resulting in twelve plans. DRR images of the anterior‐posterior (AP) and left‐right (LR) fields were generated and uploaded in iView and PIPSpro. A sample of the AP and LR DRRs of the Alderson RANDO phantom are shown in Fig. 2.

The linac isocenter was positioned at the prescription point using the phantom's reference markers, and the room lasers and portal images were acquired and registered on corresponding DRRs.

Also, in this case, all registrations (including the segmentation phase) were repeated three times, and the mean value and the standard deviation for translation and rotation parameters were evaluated.

For the statistical analysis, a multivariate analysis of variance (MANOVA) was used. Possible differences in registration algorithms, TPSs used for DRR generation and CT studies slice thicknesses were performed using Statistical Analysis Systems (SAS Institute Inc., Cary, NC) (SAS/STAT module version 9.1). Two separate analyses were conducted for AP and LR images. A *p* value <0.05 was considered statistically significant.

## III. RESULTS

### A.1 Intrinsic accuracy of the registration software

The results reported in Table 1 show the mean differences, $\bar{\Delta x},\bar{\Delta y}$, and $\bar{\Delta \alpha }$, between the registration parameters and the a priori known values.

In all cases $\bar{\Delta x},\bar{\Delta y}$ and $\bar{\Delta \alpha }$ and σ(Δx),σ(Δy) and σ(Δα) are less than 1 pixel and 1°, thus proving the good intrinsic accuracy, repeatability and equivalence of the tested algorithm.

### A.2 Overall accuracy

**Figure 3:**
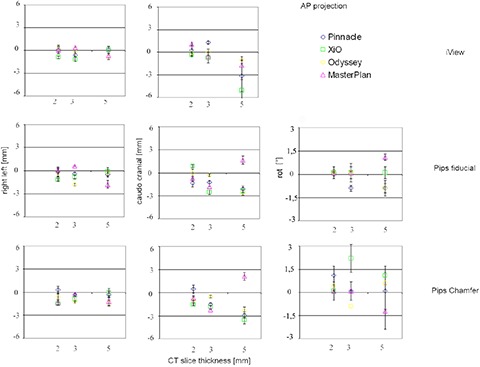
AP field overall registration accuracy. Translation and rotation registration values of the tested software are plotted against CT slice thickness. No rotation with iView software was obtained.

The overall accuracy is estimated by the registration parameters of the tested software for both AP and LR fields. The results relative to the AP field are reported in Fig. 3. Translations are along the y‐ and x‐axes, namely patient caudo‐cranial and right‐left directions, while the rotation is around the z‐axis (IEC61217 patient coordinate system^(^ ^10^ ^)^).

The overall accuracy along the right‐left axis is less or equal to 1 mm for almost all the 36 registrations performed. Results along the caudo‐cranial direction show a dependence statistically significant on CT slice thickness (p<0.0001) – the parameter's distribution spreads more with some values above 3 mm. This effect, related to an under‐sampling of the structures involved in the registration procedure, results in an inaccurate fiducial points localization or bone segmentation and, therefore, in an inaccurate caudo‐cranial translation parameter estimate.

The statistical analysis doesn't show any significant difference among the considered algorithms (p=0.1104).

When iView software is used, rotations are always 0, while values less than or equal to 1° are evaluated in almost all cases using both semiautomatic methods; a dependence on CT slice thickness is evident also in this case.

This suggests that when a manual registration software is used to align two images presenting only slight displacements, the user is led to involve translations exclusively in the registration process rather than both rotation and translations.

**Figure 4:**
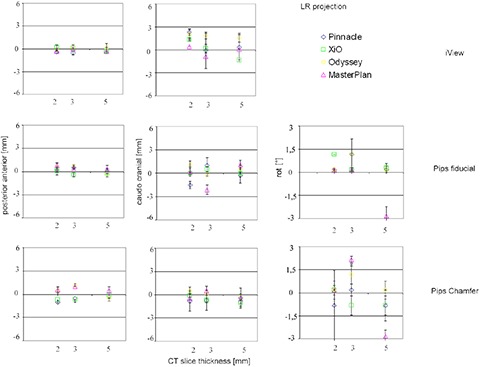
LR field overall registration accuracy. Translation and rotation registration values of the tested software are plotted against CT slice thickness. No rotation with iView software was obtained.

Results relative to LR field are reported in Fig. 4. Translations are along z‐ and y‐axes, postero‐anterior and caudo‐cranial directions, while the rotation is around the x‐axis.

The overall accuracy along the posterior‐anterior direction was equal to or less than 1 mm for all the 36 registrations performed.

Any significant difference (p=0.3389) among the considered CT studies was obtained while a difference among the registration software was observed (p=0.0153). Differences in bone segmentation of LR DRRs generated using 2 mm, 3 mm and 5 mm CT slice thicknesses are, in fact, less evident than those observed for AP DRRs. The overlapping of symmetrical structures and the scarcity of identification detail along the caudo‐cranial direction, produce an image with a poorer quality than that available in the AP field. These effects cause the manual segmentation procedure to extract almost the same structures even when thin CT slices are used and, therefore, makes the caudo‐cranial translation estimate to be always the same.

Rotations resulting from lateral field registrations are larger and more spread out than those obtained for the AP field. The same considerations previously done for iView and PIPSpro comparison hold true in this case as well.

The overall accuracy seems to hinder also the effect of the TPSs used for DRR generation (p=0.0013 for AP and p=0.0015 for LR).

## IV. DISCUSSION

In many radiotherapy clinics, geometric patient setup uncertainties are measured and corrected by registering two orthogonal DRRs on corresponding portal images. When such an approach is followed, the accuracy of patient setup depends on the accuracy of the registration software used.

The aim of this work was to evaluate and compare the accuracy of iView and PIPSpro.

The intrinsic accuracy was tested using a synthetic phantom. The two software products use different registration approaches: the first proposes a manual method while, in the second, two semi‐automatic techniques are provided. The different approaches do not lead to different results. In fact, as reported in Table 1, the differences between the registration parameters and the a priori known values are less than 1 pixel and 1°, for all the considered algorithms.

The overall accuracy was studied using DRRs and the portal images of an Alderson RANDO phantom. The detection of the corresponding fiducial points and structures during the manual segmentation phase is obviously influenced by the image quality that causes the registration accuracy and repeatability to worsen. In fact, even though the iView and PIPSpro registration approaches are different, all methods involve the user in locating corresponding structures in DRRs and portal images.

When AP images are considered, results along the caudo‐cranial direction show a significant dependence on CT image slice thickness. The coarser sampling of bone structures, caused by larger CT slice thickness, produces an inaccurate segmentation and, therefore, a less accurate and repeatable caudo‐cranial translation parameter estimate. On the contrary, no significant difference among the registration parameters evaluated using different CT studies was obtained for LR images. Differences in bone anatomy description of the LR DRRs generated using different CT slice thickness are less evident. In this case the symmetrical structures overlapping (e.g., femoral heads), together with the scarcity of useful bone details along the caudo‐cranial direction, significantly reduce the amount of reliable data in this direction. These effects suggest that much more trust should be placed on the caudo‐cranial translation parameter evaluated using the AP projection. The poorer quality of the LR DRRs and the proximity of the segmented structures or anatomical markers to the rotation axis also make the rotation parameter estimate less accurate than that obtained for AP images, as shown by the larger and widespread results reported in Fig. 3 with respect to Fig. 4.

The TPSs used to generate the AP and LR DRR images seem also to affect the registration parameter estimate. Although differences among the image quality of the DRRs were observed, this affects the DRR segmentation less significantly than the CT slice thickness.

Significant differences among the registration software, which may be attributable to the poor DRR image quality, were observed for LR images. In addition, the registration accuracy data along cranio‐caudal direction show a different behavior for iView and PIPSpro. In particular, especially for larger slices, iView data show larger standard deviation, while PIPSpro chamfer matching and fiducial point data show larger mean values.

This effect could be explained in the following manner: whereas the two PIPSpro algorithms are semi‐automatic, iView enables a manual registration by asking the operator to choose the best match moving a DRR template on a portal image. For this reason, when DRRs generated from larger CT slices are considered, the operator, aware of the poor DRR resolution, is led to choose different matching positions, thus producing a wider distribution of registration parameters. When the two PIPSpro algorithms are used, the operator has to outline structures or fiducial points on both DRRs and portal images and, based on these data, the software automatically finds the registration parameters. For this reason a more repeatable outcome and, therefore, a less widely spread registration parameter distribution will be obtained. However, it also means that, when the identification of anatomical structures in DRR is affected by the CT slice thickness, the software output could results in a systematic mismatch.

## V. CONCLUSIONS

The results obtained with iView and PIPSpro are nearly equivalent. The overall accuracy is almost the same, except for the rotation parameters estimate where the semi‐automatic methods seem to be more affected by DRR image quality than the manual approach. The results of the registrations can be considered reliable only if thin CT slices are considered. An effect on the registration accuracy imputed to the DRRs' generator was also noted.

## ACKNOWLEDGEMENTS

The author would like to thank Dr. Francesco Meucci and Dr. Lucia Paladini for their help in collecting data, and Jay Natelle for carefully reviewing the manuscript.
